# Glycolytic Genes Predict Immune Status and Prognosis Non-Small-Cell Lung Cancer Patients with Radiotherapy and Chemotherapy

**DOI:** 10.1155/2023/4019091

**Published:** 2023-04-17

**Authors:** Tianye Zhao, Jingjing Shao, Jia Liu, Yidan Wang, Jia Chen, Song He, Gaoren Wang

**Affiliations:** ^1^Nantong University Medical College, 226006, China; ^2^Department of Radiation Oncology, Nantong Tumor Hospital, Affiliated Tumor Hospital of Nantong University, 226006, China; ^3^Cancer Research Center Nantong, Nantong Tumor Hospital, Affiliated Tumor Hospital of Nantong University, 226006, China; ^4^Department of Radiology, Affiliated Hospital of Nantong University, 226006, China; ^5^Department of Oncology, Nantong Tumor Hospital, Affiliated Tumor Hospital of Nantong University, 226006, China

## Abstract

**Background:**

Non-small-cell lung cancer (NSCLC) is a major health problem that endangers human health. The prognosis of radiotherapy or chemotherapy is still unsatisfactory. This study is aimed at investigating the predictive value of glycolysis-related genes (GRGs) on the prognosis of NSCLC patients with radiotherapy or chemotherapy.

**Methods:**

Download the clinical information and RNA data of NSCLC patients receiving radiotherapy or chemotherapy from TCGA and geo databases and obtain GRGs from MsigDB. The two clusters were identified by consistent cluster analysis, the potential mechanism was explored by KEGG and GO enrichment analyses, and the immune status was evaluated by estimate, TIMER, and quanTIseq algorithms. Lasso algorithm is used to build the corresponding prognostic risk model.

**Results:**

Two clusters with different GRG expression were identified. The high-expression subgroup had poor overall survival. The results of KEGG and GO enrichment analyses suggest that the differential genes of the two clusters are mainly reflected in metabolic and immune-related pathways. The risk model constructed with GRGs can effectively predict the prognosis. The nomogram combined with the model and clinical characteristics has good clinical application potential.

**Conclusion:**

In this study, we found that GRGs are associated with tumor immune status and can assess the prognosis of NSCLC patients receiving radiotherapy or chemotherapy.

## 1. Introduction

Lung cancer is one of the most common malignancies worldwide and the leading cause of cancer-related deaths. According to statistics, the number of lung cancer cases and deaths worldwide in 2021 was about 2.2 million and 1.6 million, which is a major public health problem seriously endangering human health [1]. Non-small-cell lung cancer (NSCLC) is the main pathological type of lung cancer, which accounts for 80%-85% [2]. Although immunotherapy and targeted therapy have made significant progress in recent years, chemotherapy and radiotherapy are still the main treatment for patients with advanced NSCLC [3, 4]. At present, the 5-year overall survival rate of NSCLC patients is only 20%. In the past, the benefits of cancer treatment development are limited and unsatisfactory [2]. Therefore, more prognostic biomarkers are needed to assess the prognosis of patients and establish the corresponding individualized treatment.

Tumor metabolic reprogramming is one of the main characteristics in the process of tumorigenesis and progression, including aerobic glycolysis (Warburg effect), lipid and protein synthesis, and enhanced glutamine metabolism [5]. The change of glycolysis is the main feature, which means tumor cells still have active glycolytic activity in the environment of sufficient oxygen and produce large amounts of high lactate metabolites. This change can lead to tumor proliferation, enhanced ability to invade, and metastasize [6]. Previous studies have made it clear that aerobic glycolysis is closely relevant to the resistance of tumor radiotherapy or chemotherapy [7, 8]. For example, inhibiting pkm2-mediated aerobic glycolysis can reverse 5-FU resistance in colon cancer [9]. Long noncoding RNA urothelial carcinoma-associated protein 1 regulates radiation resistance through hexokinase 2/glycolysis pathway in cervical cancer [10]. These related studies suggest that understanding the mechanism of glycolysis may help us find potential prognostic markers. Tumor immune microenvironment (time) also plays an important role in chemotherapy and radiotherapy resistance and significantly affects the prognosis of tumor patients [11–13]. In addition, tumor immune microenvironment is also significantly related to glycolysis. Tumor cells use enough energy produced by glycolysis to change their immune microenvironment and inhibit the activation of immune cells and tumor inhibition, leading to tumor immune escape [14–16]. Metabolic competition between tumor cells and immune cells will further promote tumor immunosuppression [17]. Therefore, the combined analysis of glycolysis and immune status of patients will help us further understand the prognosis of NSCLC patients with radiotherapy or chemotherapy.

In this study, we specifically analyzed the glycolysis-related genes (GRGs) and studied the effect of glycolysis on the survival and immune status of NSCLC patients undergoing radiotherapy or chemotherapy. In addition, we also build a risk scoring model based on GRGs. The model showed good prognostic ability in NSCLC patients and different clusters. This study can help us better explore the potential mechanism of poor prognosis of NSCLC patients with radiotherapy or chemotherapy and provide new ideas for better personalized treatment.

## 2. Materials and Methods

### 2.1. Data Collection

Download the data set of LUAD and LUSC from TCGA (https://portal.gdc.cancer.gov/) and GEO (https://www.ncbi.nlm.nih.gov/geo/) databases as patient data of non-small-cell lung cancer, including gene expression profile of mRNA sequencing samples and clinical prognosis information. Inclusion criteria are as follows: (1) have complete clinical information and gene expression matrix; 2) are not repeated tumor samples; 3) received radiotherapy or chemotherapy: 116 samples are in TCGA database as training cohort. 49 samples are in GSE42127 dataset in GEO database as validation cohort. 289 GRGs were identified from the “HALLMARK GLYCOLYSIS” “REACTOME GLYCOLYSIS” “KEGG GLYCOLYSIS GLUCONEOGENESIS” gene sets in the MsigDB database.

### 2.2. Cluster Analysis

First, we use the R package “Survival” to integrate gene expression data, survival time, and status and use the univariate Cox regression method to obtain 11 prognosis-related GRGs. Then, use the R package “Consensus Cluster Plus” to perform cluster analysis based on these 11 genes. Determine the optimal number of clusters *K* = 2 through the cumulative distribution curve graph.

### 2.3. Differential Genes and Functional Analyses

The differential genes in the two clusters were evaluated using the R package “t.test” function, and the differential genes with adjust *p* value <0.05 and |logFC| > 1.5 were selected. Next, we used the gene annotation of the KEGG pathway obtained by the KEGG rest API and the GO annotation of the genes in the R package “http://Org.Hs.eg.db” and performed the enrichment analysis on the differential genes obtained by the R package “Cluster Profiler”, to analyze functional differences between clusters.

### 2.4. Immune Analyses

Using the R package IOBR, the ESTIMATE method was selected to calculate the stroma, immune, and estimate scores of each group of samples. The TIMER and quanTIseq methods were selected to calculate the immune infiltrating cell score of each group of samples.

### 2.5. Risk Score Model

Using the R package “Glmnet”, the survival time, survival status, and expression data of 11 GRGs were integrated, and the Lasso-Cox method was used for regression analysis. We chose the minimum lambda value of 0.0824 to obtain the optimal model. Based on this, we obtained 7 genes (ACSS1, ERO1A, GPC4, MERTK, PKP2, TXN, and ZNF292) and established a risk scoring model using their expression: risk score = −0.0308 × ACSS1 + 0.1205 × ERO1A-0.2470 × GPC4-0.0063 × MERTK + 0.0497 × PKP2 + 0.0584 × TXN-0.0491 × ZNF29. The samples were divided into high- and low-risk groups according to the obtained risk scores. Kaplan-Meier survival analysis was used to analyze the difference in overall survival between the two groups. Time-dependent ROC curve analysis was used to evaluate the prognostic predictive value of this risk model. Finally, a multivariate survival regression nomogram was constructed to evaluate the prognostic significance of risk model, tumor stage, and other characteristics in these samples.

### 2.6. Correlation Pathway Analysis

The enrichment score of each sample in the gene set was calculated from the Gene Set Variation Analysis (GSVA) using the R package, and the hallmark gene sets were downloaded from the Molecular Signatures Database to evaluate the relevant pathways and molecular mechanisms.

### 2.7. Statistical Analysis

Statistical analysis was performed using R software (version 4.0.5) and GraphPad Prism (version 8. 0. 1). Survival analysis was done using the Kaplan-Meier method. Differences between the two groups were determined by Student's two-tailed *t*-test. *p* < 0.05 was considered a significant difference.

## 3. Results

### 3.1. Two Clusters Identified Based on GRGs

**Figure 1:**
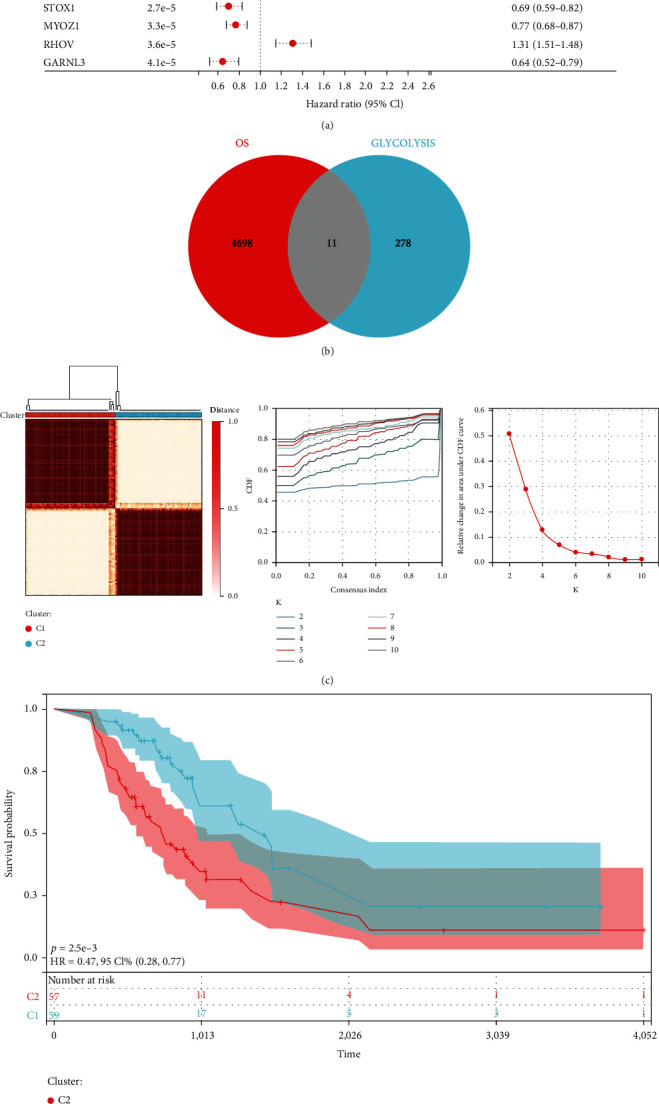
Survival-related GRGs were screened, and concordant cluster analysis was performed. (a) Top 10 genes screened in the TCGA dataset (ranked by *p* value). (b) Venn diagram showing 11 prognostic GRGs identified in survival genes. (c) *K* = 2 is clearly identified as the best clustering value. (d) Survival curves of the two clusters.

In this study, we selected the NSCLC dataset of TCGA and filtered out a total of 116 patients with radiotherapy or chemotherapy. Based on this dataset, univariate Cox regression was performed. The analysis yielded 4709 genes closely associated with prognosis. The top 10 prognosis-associated genes are presented in Figure 1(a) (ranked by *p* value). The Venn diagram indicated that 11 prognostic glycolysis genes were identified among these prognosis genes (ACCSS1, ERO1A, GPC4, PKP2, TXN, MERTK, ZNF292, ALDH3B2, PAM, and RRAGD) (Figure 1(b)). The patients in the dataset were divided into two groups using consistent cluster analysis. 59 patients were clustered into cluster 1, and 57 patients were clustered into cluster 2 (Figure 1(c)). C1 represents the low-expression cluster of GRGs, while C2 represents the opposite. Patients of cluster 2 had significantly worse overall survival than cluster 1 (*p* < 0.001; Figure 1(d)). These results suggest that glycolytic genes segregate NSCLC patients who have received chemotherapy or radiation into two clusters with different overall survival.

### 3.2. Differential Gene and Functional Analyses of Two Clusters

**Figure 2:**
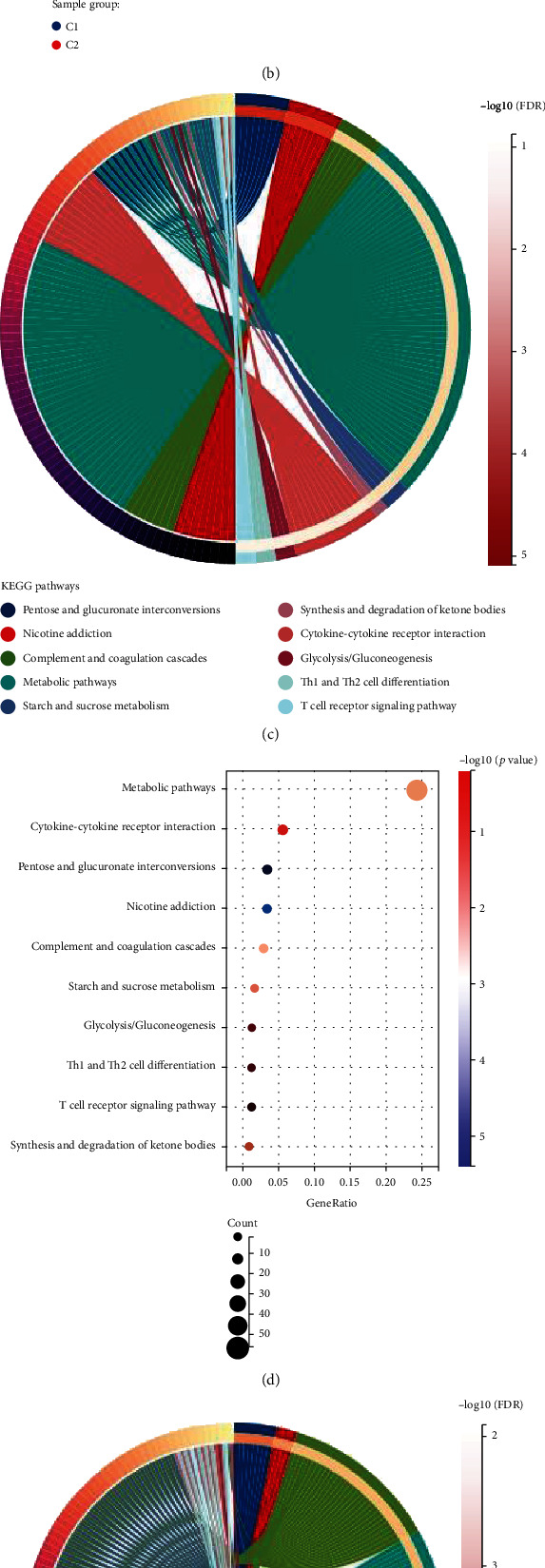
Differential gene and functional analysis of the two clusters. (a, b) Volcano plots and heat maps showing differential genes between the two clusters. (c, d) Network bubble and circle plots showing GO enrichment analysis results. (e, f) Network bubble and circle plots showing KEGG enrichment analysis results.

To further probe the underlying mechanism of the difference in survival of these two clusters, we identified their differential genes and performed functional analysis. There were 3669 significantly differential genes, of which 1766 genes were upregulated in cluster 2 compared to cluster 1, and 1903 genes were downregulated (Figures 2(a) and 2(b)). KEGG enrichment analysis showed that the differential genes were enriched in biological functions such as glucose metabolism, immunity, and nicotine addiction (Figures 2(c) and 2(d)). GO enrichment analysis also showed that differential genes were enriched in biological processes such as immunity and glucose metabolism (Figures 2(e) and 2(f)). These results indicate that the expression of glycolytic genes is closely related to the immune biological function. The abnormal immune function caused by these genes may be a contributing factor to the poor prognosis of patients with non-small-cell lung cancer after radiotherapy and chemotherapy.

### 3.3. Immune Analyses

**Figure 3:**
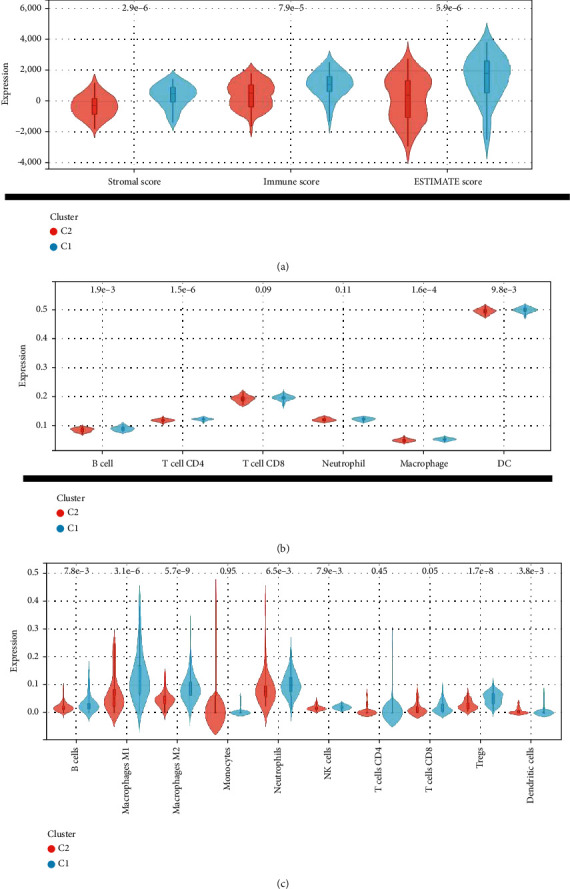
Immunoassays in two clusters. (a) Stroma, immune, and ESTIMATE scores. (b) TIMER immune score. (c) quanTIseq score.

Next, we performed an immune analysis of patients in both molecular clusters to explore immune differences between them. The ESTIMATE algorithm showed that patients in cluster 1 had significantly higher stromal score compared to cluster 2 (*p* < 0.001), immune score (*p* < 0.001), and ESTIMATE score (*p* < 0.001; Figure 3(a)). The TIMER algorithm indicated that B cells (*p* < 0.001), CD4 T cells (*p* < 0.001), and macrophages (*p* < 0.001) in cluster 1 and DC cells (*p* < 0.001) were significantly higher than in cluster 2 (Figure 3(b)). In addition, the quanTIseq algorithm also showed that B cells (*p* < 0.001), macrophages M1 (*p* < 0.001), B cells (*p* < 0.001), macrophages M1 (*p* < 0.001), macrophages M2 (*p* < 0.001), neutrophils (*p* < 0.001), NK cells (*p* < 0.001), CD8 T cells (*p* = 0.05) (*p* < 0.001), Tregs (*p* < 0.001) and DC cells (*p* < 0.001) was more abundant (Figure 3(c)). These results suggest that there are significant immune differences between the two subtypes.

### 3.4. Establishment of the Risk Score Model Based on GRGs

**Figure 4:**
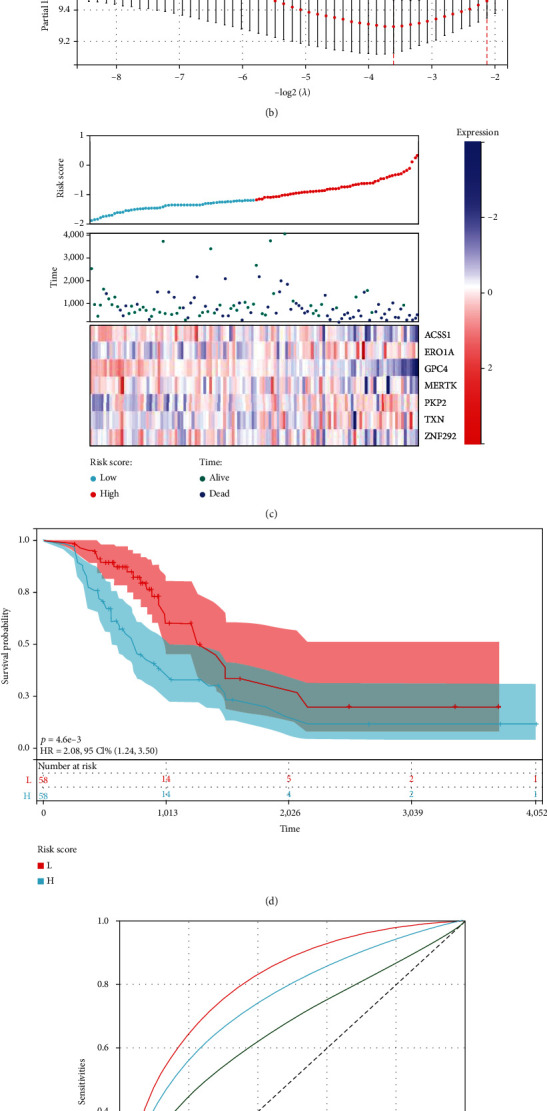
Construction of a risk model for NSCLC patients who have received chemotherapy or radiotherapy. (a) Lasso analysis, the analysis result with the smallest *λ* value, is selected. (b) Survival status: distribution of risk scores and heat map of candidate gene expression for patients in high-risk and low-risk groups. (c) Survival curves of the two groups of patients. (d) Time-dependent ROC curve of the risk model.

To build a more accurate prognosis model, we used Lasso regression analysis to screen glycolysis prognosis genes and selected 7 genes (ACSS1, ERO1A, GPC4, PKP2, TXN, MERTK, and ZNF292) with *λ* = 0.09 as candidate genes (Figures 4(a) and 4(b)). Based on the results, these 7 genes were identified to construct a risk model. The patients were divided into high- and low-risk groups by this risk model, and it was observed that with the increase of the risk score, the survival rate of the patients decreased significantly (Figure 4(c)). The results of Kaplan-Meier survival analysis showed that the overall survival of the patients in the low-risk group was significantly better than that of the patients in the high-risk group.  Figure 4(d); *p* < 0.001). ROC curve analysis showed that the AUC values for 1-year, 3-year, and 5-year survival rates were 0.82, 0.75, and 0.72, respectively. (Figure 4(e)), indicating that the constructed risk model exhibited accurate predictive power over a 5-year period.

### 3.5. Prognostic Value of Risk Score Models in Different Clinical Clusters

**Figure 5:**
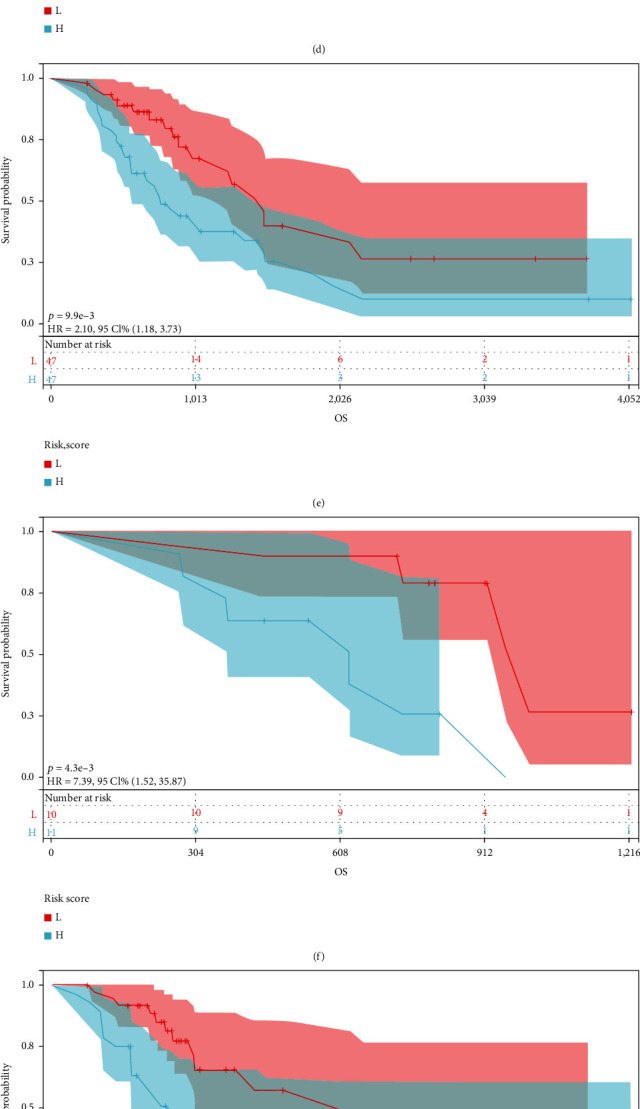
Association of risk scores and clinical characteristics. (a–j) Independence analysis of risk models. Survival curves of patients regrouped according to age (a, b), smoking history (c, d), T stage (e, f), N stage (g, h), and clinical stage (i, j).

To further evaluate the role of the risk model in clinical application, we analyzed the prognostic value of the model for patients with different clinical characteristics (age, smoking, T stage, N stage, and clinical stage). The prognosis of the high-risk score group was always worse than that of the low-risk score group (Figures 5(a)–5(j)). In conclusion, the prognostic value of this risk model for NSCLC patients with chemotherapy or radiotherapy was not perturbed by other clinical characteristics.

We next used univariate and multivariate Cox regression to analyze the association between risk scores, other clinical characteristics, and prognosis in NSCLC patients with chemotherapy or radiotherapy. Both N stage (HR = 2.294, *p* = 0.001) and tumor stage (HR = 2.232, *p* = 0.004) were significantly associated with prognosis. Multivariate Cox analysis showed that the risk score (HR = 4.191, *p* < 0.001) was the highest risk factor for patients receiving overexposure independent prognostic factors in chemotherapy-treated NSCLC patients (Table 1). Taken together, these results suggest that this risk model has good prognostic value in NSCLC patients with chemotherapy or radiotherapy.

### 3.6. Risk Score Correlates with Activity of Chemotherapy and Radiotherapy Resistance-Related Pathways

**Figure 6:**
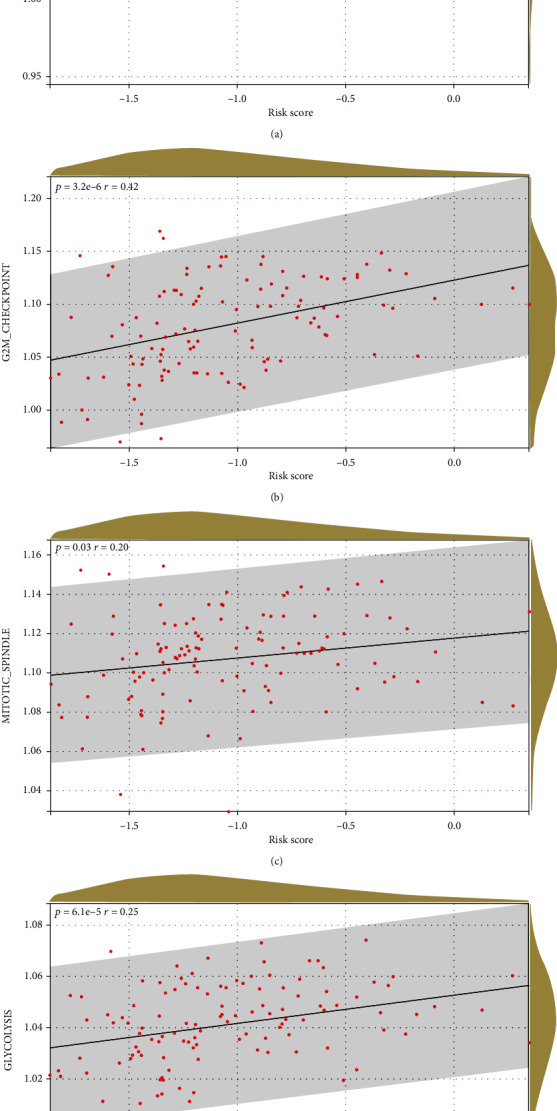
Association of risk scores with DNA repair (a), G2M checkpoint (b), mitotic spindle (c), and glycolysis (d) pathways.

Thereafter, the relationship between the risk score and chemotherapy and radiotherapy resistance-related pathways was assessed. Using the ssGSEA algorithm, we found that the higher the risk score, the greater the risk of DNA repair (*p* < 0.001, *R* = 0.32), and G2M checkpoint (*p* < 0.001, *R* = 0.42), mitotic spindle (*p* = 0.03, *R* = 0.20), and glycolytic (*p* < 0.001, *R* = 0.25) pathways were more active (Figures 6(a)–6(d)). These results showed that with increasing risk score, pathway activity associated with chemotherapy and radiotherapy resistance also increased, suggesting a poor prognosis.

### 3.7. Construction of a Nomogram

**Figure 7:**
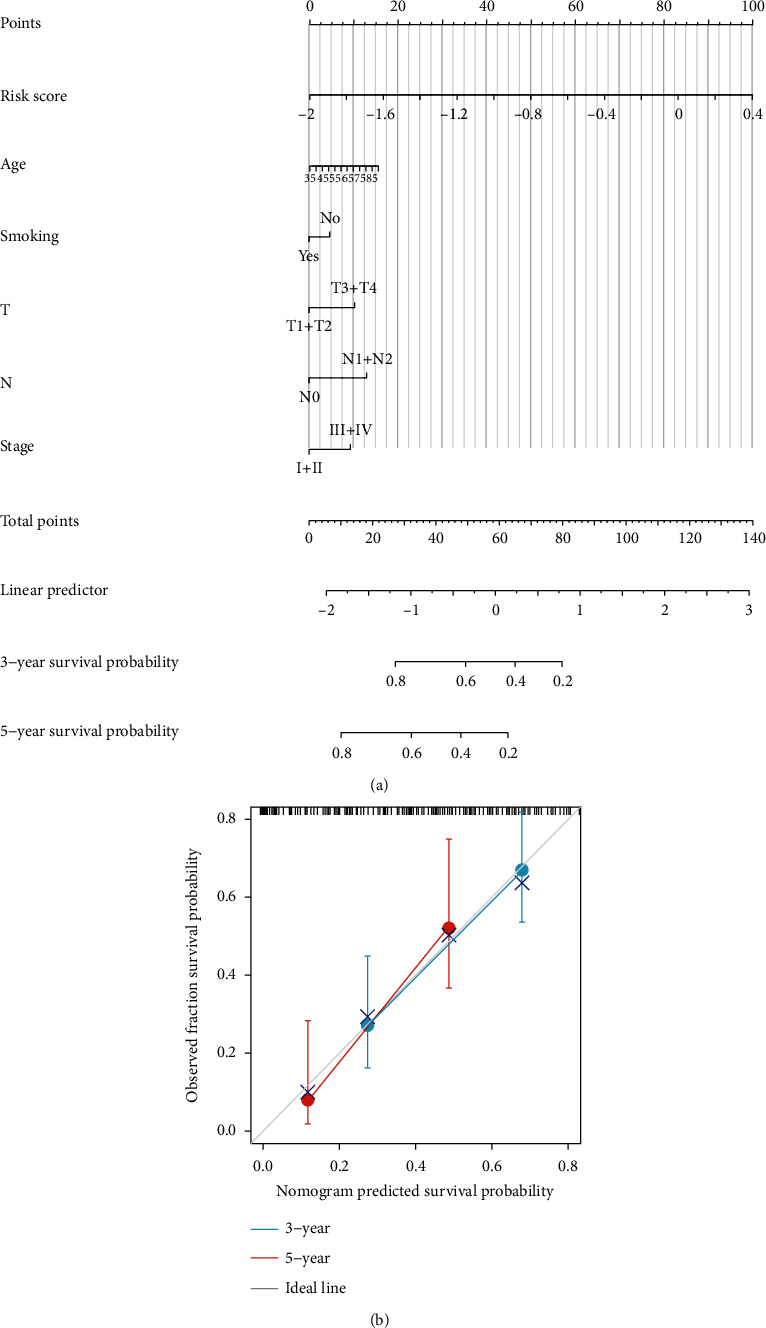
Nomogram construction and calibration. (a) Nomogram of composite risk score and clinical characteristics. (b) Nomogram calibration at 3 and 5 years.

Thereafter, we constructed nomograms integrating risk models and clinical characteristics to provide a quantitative method for predicting 3- and 5-year OS probabilities in NSCLC patients with chemotherapy or radiotherapy, which can then be used in clinical practice. Based on multivariate Cox regression, as a result of the analysis, the nomogram integrated clinicopathological features and risk scores (Figure 7(a)). The c-index value of the nomogram was 0.721, and the 3- and 5-year calibration curves were in good agreement with the standard curve, indicating that the model provided. The predictive performance at an effective level was obtained (Figure 7(b)). Therefore, this risk score-based nomogram can be used in clinical practice to predict the prognosis of NSCLC patients with chemotherapy or radiotherapy.

### 3.8. Clinical Prognostic Value of Risk Scoring Models in a Validation Cohort

**Figure 8:**
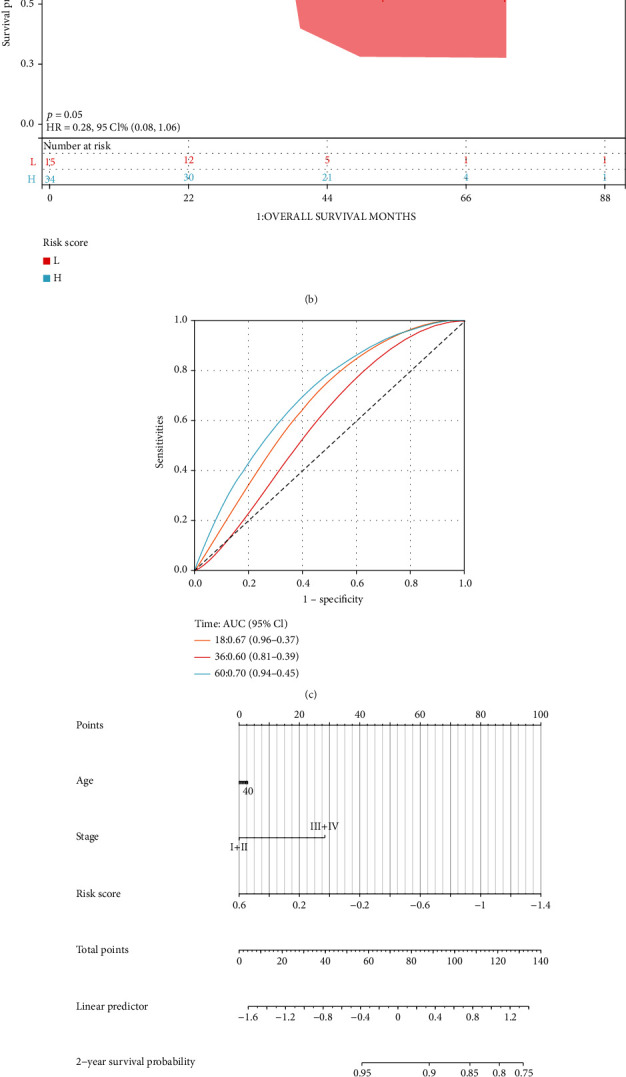
Validate the constructed risk model in a validation queue. (a) Survival status, risk score distribution, and heat map of candidate gene expression for the validation cohort. (b) Survival curves of patients in high-risk and low-risk groups in the validation cohort. (c) Time-dependent ROC curves of the risk model in the validation cohort. (d) Nomogram of composite risk scores and clinical characteristics in the validation cohort. (e) Nomogram calibration at 3 and 5 years.

Finally, we independently used the patients receiving chemotherapy in the GSE42127 dataset as the validation set of the risk model. The expression of seven genes was shown by a heat map (Figure 8(a)). Kaplan-Meier survival analysis results also showed that patients with higher risk scores had a worse prognosis. (Figure 8(b); *p* = 0.05). ROC curve analysis showed that the risk score had the best prediction effect at 5 years (Figure 8(c)). The constructed nomogram also proved that the risk score model has considerable value in clinical prognostic work (Figures 8(d) and 8(e)).

## 4. Discussion

Non-small-cell lung cancer is one of the most emerging and deadly cancers worldwide, with a markedly poor prognosis [2]. Radiotherapy and chemotherapy are the main treatments for patients with advanced NSCLC, but these treatments often fail to achieve satisfactory results [3, 4]. Therefore, there is an urgent need to find potential prognostic markers for NSCLC patients with radiotherapy or chemotherapy. Although there have been many studies on NSCLC prognostic markers, most of them are not focused on radiotherapy or chemotherapy patients and have not penetrated into radiotherapy resistance and chemotherapy resistance. High activity of glycolysis and abnormal immune microenvironment are two important hallmarks of cancer [18] and are closely related to radioresistance and chemoresistance [7, 8]. In this study, we screened out the prognostic GRGs and divided the patients into two clusters according to their expression levels. They have different clinical prognostic values and immune scores. These immune scores include T cell, B cell, neutrophil, and macrophage activity scores, which represent the immune microenvironment state of tumor. They can also be used as a reference for immunotherapy and can also evaluate the disease progress and comprehensive treatment prognosis of tumor patients [19]. In addition, we also constructed a risk score model based on GRGs, which can accurately predict the prognosis of NSCLC patients undergoing radiotherapy or chemotherapy. Our findings may provide new ideas for the development of treatment regimens for NSCLC patients.

First, we selected NSCLC patients who had received radiotherapy or chemotherapy in the TCGA and GEO databases as research subjects and screened out 10 GRGs (ACSS1, ERO1A, GPC4, PKP2, TXN, MERTK, ZNF292, ALDH3B2, PAM, RRAGD), and two clusters were identified based on their expression levels, which were significantly different in overall survival. We then performed differential gene and functional enrichment analysis on these two clusters to explore the underlying mechanisms of this survival difference. KEGG and GO enrichment analyses showed that the difference in immune and metabolic functions may mediate the effect of GRGs on the prognosis of patients with NSCLC after radiotherapy or chemotherapy. Therefore, we used ESTIMATE, TIMER, and quanTIseq scores to evaluate the immune infiltration of the two clusters. It has been shown to be closely related to the efficacy of radiotherapy or chemotherapy in NSCLC. The results showed that the subgroup with higher expression of GRGs had lower immune scores, which may be closely related to poor prognosis.

Based on the above results, we used 7 genes (ACSS1, ERO1A, GPC4, PKP2, TXN, MERTK, and ZNF292) to construct a risk model to predict the prognosis of NSCLC patients with radiotherapy or chemotherapy. ACSS1 and ERO1A are highly expressed in tumors and can promote tumor progression metabolic changes associated with cancer cell survival [20–23]. PKP2, TXN, and ZNF292 are also abnormally expressed in tumors and induce radioresistance of tumor cells [24–27]. GPC4 can activate the Wnt/*β*-catenin pathway and its downstream targets to increase 5-fluorouracil (5-FU) resistance and cell stemness in pancreatic cancer [28]. MERTK can inhibit the immune effect of the body against tumors through the inflammatory pathway and PD-1 signaling axis, as well as regulating the functions of various immune cells [29]. MERTK inhibitors have been confirmed to be used in combination with radiotherapy or chemotherapy in glioma, NSCLC, head and neck squamous cell carcinoma, and other tumors to achieve better efficacy [30–32]. In addition, ERO1A, PKP2, and MERTK have been proved to promote the progress and drug resistance of NSCLC by enhancing the activation of tumor and PI3K, EGFR, and other signal pathways [33–35]. In this study, we identified the good prognosis prediction effect of this risk model and confirmed this prediction effect using chemotherapy patients in the GSE42127 dataset as a validation cohort. This may help the clinical treatment of NSCLC patients with chemotherapy or radiotherapy and provide potential targets for individualized treatment.

Although our study provides a risk model constructed with GRGs that has a good predictive effect on the prognosis of NSCLC patients with chemotherapy or radiotherapy, there are still many limitations. First, all data in our study are publicly available retrospective samples, and a certain number of prospective samples need to be included to confirm our results. Second, we only focus on clinical prognosis and do not dig deep into specific molecular mechanisms. Third, our research is a bioinformatics study, and there is a lack of specific basic experiments to verify.

## 5. Conclusion

In conclusion, this study clustered NSCLC patients with chemotherapy or radiotherapy into two clusters based on GRGs. Functional analysis and immune scores showed that high glycolytic activity can lead to suppressed immune status and poor prognosis. At the same time, we also established a corresponding risk scoring model, hoping to provide new ideas and theoretical support for clinical treatment.

## Figures and Tables

**Table 1:** Univariate and multivariate analyses of risk scores and characteristics.

Characteristics	Total (*N*)	Univariate analysis		Multivariate analysis	
		Hazard ratio (95% CI)	*p* value	Hazard ratio (95% CI)	*p* value
Risk score	116	5.023 (2.856-8.835)	**<0.001**	4.191 (2.346-7.485)	**<0.001**
Age	116	0.998 (0.972-1.023)	0.848		
Smoking	116				
No	73	Reference			
Yes	43	1.035 (0.624-1.717)	0.893		
T stage	115				
T1 + T2	94	Reference			
T3 + T4	21	1.636 (0.851-3.145)	0.140		
N stage	114				
N0	65	Reference			
N1 + N2	49	2.294 (1.384-3.803)	**0.001**	1.751 (0.987-3.105)	0.055
Stage	114				
I + II	83	Reference			
III + IV	31	2.232 (1.288-3.868)	**0.004**	1.482 (0.822-2.670)	0.191

## Data Availability

The datasets used in this study are available in online databases. They come from the TCGA and GEO databases.
